# Anti-Alzheimer’s Studies on β-Sitosterol Isolated from *Polygonum hydropiper* L.

**DOI:** 10.3389/fphar.2017.00697

**Published:** 2017-10-06

**Authors:** Muhammad Ayaz, Muhammad Junaid, Farhat Ullah, Fazal Subhan, Abdul Sadiq, Gowhar Ali, Muhammad Ovais, Muhammad Shahid, Ashfaq Ahmad, Abdul Wadood, Mohamed El-Shazly, Nisar Ahmad, Sajjad Ahmad

**Affiliations:** ^1^Department of Pharmacy, University of Malakand, Chakdara, Pakistan; ^2^Department of Pharmacy, University of Peshawar, Peshawar, Pakistan; ^3^Cancer Biology Lab, Department of Biotechnology, Quaid-i-Azam University, Islamabad, Pakistan; ^4^Department of Pharmacy, Sarhad University of Science and Information Technology, Peshawar, Pakistan; ^5^Department of Biochemistry, Abdul Wali Khan University, Mardan, Pakistan; ^6^Department of Pharmacognosy and Natural Products Chemistry, Faculty of Pharmacy, Ain-Shams University, Cairo, Egypt

**Keywords:** *Polygonum hydropiper*, Alzheimer’s disease, β-sitosterol, cholinesterases, antioxidant, shallow water maze, Y-maze, molecular docking

## Abstract

The family Polygonaceae is known for its traditional use in the management of various neurological disorders including Alzheimer’s disease (AD). In search of new anti-AD drugs, β-sitosterol isolated from *Polygonum hydropiper* was subjected to *in vitro, in vivo*, behavioral and molecular docking studies to confirm its possibility as a potential anti-Alzheimer’s agent. The *in vitro* AChE, BChE inhibitory potentials of β-sitosterol were investigated following Ellman’s assay. The antioxidant activity was tested using DPPH, ABTS and H_2_O_2_ assays. Behavioral studies were performed on a sub-strain of transgenic mice using shallow water maze (SWM), Y-maze and balance beam tests. β-sitosterol was tested for *in vivo* inhibitory potentials against cholinesterase’s and free radicals in the frontal cortex (FC) and hippocampus (HC). The molecular docking study was performed to predict the binding mode of β-sitosterol in the active sites of AChE and BChE as inhibitor. Considerable *in vitro* and *in vivo* cholinesterase inhibitory effects were observed in the β-sitosterol treated groups. β-sitosterol exhibited an IC_50_ value of 55 and 50 μg/ml against AChE and BChE respectively. Whereas, the activity of these enzymes were significantly low in FC and HC homogenates of transgenic animals. Molecular docking studies also support the binding of β-sitosterol with the target enzyme and further support the *in vitro* and *in vivo* results. In the antioxidant assays, the IC_50_ values were observed as 140, 120, and 280 μg/ml in the DPPH, ABTS and H_2_O_2_ assays respectively. The free radicals load in the brain tissues was significantly declined in the β-sitosterol treated animals as compared to the transgenic-saline treated groups. In the memory assessment and coordination tasks including SWM, Y-maze and balance beam tests, β-sitosterol treated transgenic animals showed gradual improvement in working memory, spontaneous alternation behavior and motor coordination. These results conclude that β-sitosterol is a potential compound for the management of memory deficit disorders like AD.

## Background

Alzheimer’s disease (AD) is a common neurological disorder, characterized by gradual memory loss, cognitive dysfunctions and behavioral turbulence. There are 33.9 millions sufferers worldwide with an estimated three folds increase in disease burden by the next 40 years (Brookmeyer et al., 2007). AD is the most common cause of dementia affecting 5.3 million people in United States (Dubois et al., 2010; Alzheimer’s Association, 2015). The underlying pathophysiological changes in AD include accumulation of amyloid peptide (Aβ), highly phosphorylated tau protein, deficiency of essential neurotransmitters like ACh and free radicals induced neuronal damage (Selkoe, 2001; Reisberg et al., 2003; LaFerla et al., 2007; Minati et al., 2009). Consequently, inhibition of AChE, BChE, β-amyloid cleaving enzyme (BACE1/beta secretase), MAO and free radicals have shown promise in the development of therapeutic agents against AD (Reisberg et al., 2003; Birks, 2006). Currently, only five anti-AD drugs are clinically approved, which include donepezil, tacrine, galanthamine, and rivastigmine as cholinesterase inhibitors and memantine as a glutamatergic system modifier (Anand and Singh, 2013). However, these drugs only provide symptomatic relief, and there is a lack of curative treatment of this disease. Moreover, no anti-amyloid drug is clinically approved, although several potential compounds are currently under evaluation in clinical trials (Hardy and Selkoe, 2002; Mangialasche et al., 2010).

To take over the limitation of one-drug-one-target strategy, researchers are in constant modifications of drug molecules for the development of multi-target drugs. However, this strategy has several limitations including complex pharmacokinetic behavior, potential toxicity and poor IVIVC (Youdim and Buccafusco, 2005; Ji and Zhang, 2008). Alternatively, highly effective and safe natural product based molecules can be screened for their beneficial therapeutic utility (Ji and Zhang, 2008). In this regard, several compounds including curcumin, quercetin, myricetin, epicatechin gallate and gossypetin have been reported to inhibit Aβ formation, cholinesterases, tau proteins aggregation, free radicals and sequester metal ions (Rice-Evans et al., 1996; Greig et al., 2005; Rezai-Zadeh et al., 2005; Aggarwal et al., 2007) and these may therefore possess therapeutic implications in neurodegenerative diseases. Several flavonoids have been reported as efficient inhibitors of MAO, AChE, BChE enzymes and free radicals scavengers (Mazzio et al., 1998; Pietta, 2000; Katalinić et al., 2010; Uriarte-Pueyo and Calvo, 2011). Accordingly, natural products, especially flavonoids are promising lead compounds for developing multi-potent agents to combat AD.

Phytosterols, a subgroup of the steroids having close structure resemblance with cholesterol and are widely distributed in herbs, fungi and animals (Wright et al., 1993). Phytosterols including cholesterol represent major part of eukaryotic cellular membranes, act as secondary messengers and regulate cell signaling and physiological processes (Wright et al., 1993). β-sitosterol, a phytosterol is a major part of human diet with several health promoting and disease mitigating effects (Saeidnia et al., 2014). According to European Foods Safety Authority (EFSA), daily consumption of 1.5–2.4 g of phytosterols significantly reduce blood cholesterol level (Lees et al., 1977; Katan et al., 2003), whereas, Food and Drug Administration (FDA) recommends that consumption of diet rich in phytosterol esters significantly reduce the risk of cardiovascular diseases (FDA, 2002). Natural products and foods including vegetables, vegetable oils, fruit, berries, nuts and cereal products are known as rich sources of phytosterols (Valsta et al., 2004). Phytosterols have a long history of use in pharmaceuticals and foods and are commonly known as safe and devoid of major side effects (Sudhop et al., 2002). Several studies revealed different preliminary neuroprotective and antioxidant effects of β-sitosterol (Vivancos and Moreno, 2005; Baskar et al., 2012). In a previous study, β-sitosterol was reported to elevate the level of antioxidant enzymes in colon carcinogenesis (Baskar et al., 2012). β-sitosterol increases the action of antioxidant enzymes via stimulation of estrogen receptor/PI3-kinase-reliant pathway. The level of total glutathione subsequent to β-sitosterol therapy suggests that it might be an effective free radicals scavenger (Vivancos and Moreno, 2005). Furthermore, glucose oxidase-mediated oxidative stress and lipid peroxidation might be inhibited via incorporation of β-sitosterol into cell membrane that showed valuable effects of this compound in neurodegenerative disorders including AD (Shi et al., 2013).

The family Polygonaceae, also known as the buckwheat, smartweed or knotweed family, consists of about 1200 species and 48 genera (Heywood, 1993). In Pakistan, Polygonaceae is represented by 19 genera and 103 species (Qaiser, 2001). *Polygonum hydropiper* is used as folk remedy for the treatment of inflammation, headache, arthritis, epilepsy, colic pain, fever and infectious diseases (Sharma, 2003). It is also used in the management of insomnia, hypertension, angina helminthesis and kidney disorders (Chevallier, 1996). Several species of Polygonaceae family have been reported for their potential effectiveness in Parkinson’s disease (Chen et al., 2007), cerebral ischemia (Chan et al., 2003) and other neurodegenerative disorders (Yang et al., 2005; Liu et al., 2010). Recently solvent extracts and essential oils from *P. hydropiper* have been reported to exhibit anticholinesterase, antioxidant and gastroprotective activities (Ayaz et al., 2014a, 2015, 2017a). On this basis, in the current study the most potent fraction was subjected to extraction techniques and β-sitosterol was isolated which was subjected to various *in vitro, in vivo*, behavioral and molecular docking studies for its prospective effectiveness in the treatment of AD.

## Materials and Methods

### Chemicals and Drugs

AChE from *Electrophorus electricus* (CAS: 9000-81-1) was obtained from Sigma Aldrich, St. Loius, MO, United States, while BChE was derived from equine serum (9001-08-5) and was purchased from Sigma–Aldrich GmbH, Germany. Acetylthiocholine iodide (CAS1866-15-5) and butyrylthiocholine iodide (CAS 2494-56-6) were purchased from Sigma–Aldrich United Kingdom and Sigma–Aldrich Switzerland respectively. 5,5-Dithio-bis-nitrobenzoic acid (DTNB) (CAS 69-78-3) (Sigma–Aldrich GmbH, Germany) and galanthamine HBr *Lycoris* Sp. (CAS: 1953-04-4) (Sigma–Aldrich, France) were used in enzymes studies. Antioxidant reagents including DPPH (CAS: 1898-66-4) ABTS (CAS: 30931-67-0) were purchased from Sigma Aldrich St. Loius, MO, United States. H_2_O_2_ (batch no: A040) was obtained from Rehmat pharma Lahore, Pakistan. Potassium peroxodisulfate (LOT NO: 51240) was obtained from Labor chemikalien GmbH & Co KGD-30926 Seelze. For genotyping of transgenic animals, GF-1 tissue DNA extraction kit (Cat:GF-TD-100, Vivantis), agarose (Invitrogen CAT:75510-011, Carlsbad, CA, United States), boric acid (Serva CAT 15165, Germany), DNA Ladder (Serva CAT:15165, Germany), EDTA (Invitrogen CAT:75576-028, Carlsbad, CA, United States), ethanol (Merck CAT:26225745, Germany), ethidium bromide (Sigma CAT:E7637, United States), MgCl_2_ (Invitrogen CAT:AM9530G, Carlsbad, CA, United States), DNTPs (Promega CAT:U1515, United States), Taq polymerase (Thermo Scientific CAT: EP0402, United States), PCR primers (Thermo Scientific CAT:OIMR3610 F, OIMR3611 R), PCR grade distilled water (Thermo Scientific CAT: R0581), sucrose (Invitrogen CAT: 15503-022, Carlsbad, CA, United States), Tris EDTA solution (50X), 2XPCR Master mix (Fermentas CAT: K0171, EU), NaCl (Invitrogen CAT: 24740-011, Carlsbad, CA, United States) and tris (Invitrogen CAT: 15504-020, Carlsbad, CA, United States) were purchased from authorized dealers in Pakistan. Solvents and buffer salts used were of extra pure quality.

### Plant Selection, Identification and Isolation

**FIGURE 1:**
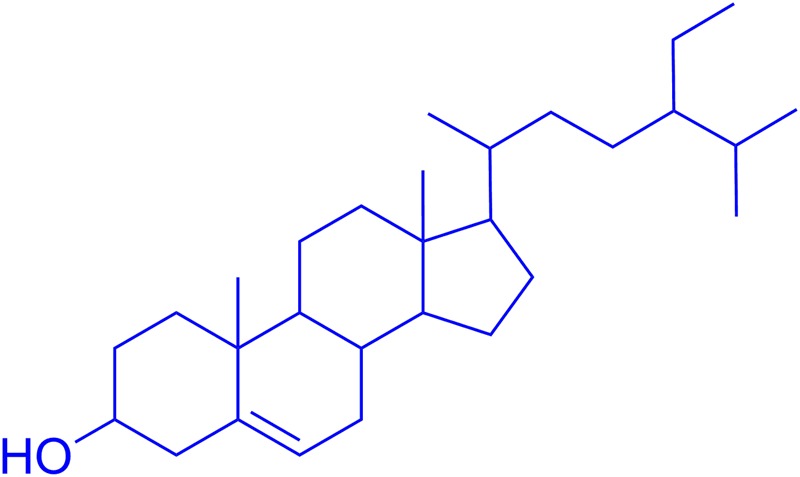
Chemical structure of β-sitosterol.

In the search for new anti-Alzheimer’s and neuroprotective drugs from Polygonacae, *P. hydropiper* L. was identified, collected and processed for fractionation as previously reported from our laboratory (Ayaz et al., 2014b, 2016). A plant specimen was deposited with voucher no H.UOM.BG.107 at the herbarium of the University of Malakand, Chakdara, Dir (L), KP, Pakistan. Solvent based fractions and saponins were initially subjected to *in vitro* anti-Alzheimer’s studies. The solvent fractions, especially chloroform and ethyl acetate were selected for the isolation of pure compounds due to their prominent activities in preliminary assays. Several compounds were isolated using gravity column chromatography eluting with *n*-haxane and ethyl acetate. The purified compounds were rotary evaporated to remove any remaining solvent. Initially, ^1^H NMR spectra was obtained to reveal the chemical structure by comparing the spectra with those reported in the literature. The ^13^C NMR spectra were used to ascertain the carbon skeleton of the compounds. The spectral data was supplemented by using mass spectrometry to confirm the structures. Among the isolated compounds, β-sitosterol was most active in the preliminary analysis and was evaluated in detail (**Figure 1**).

## *In Vitro* Studies

### Cholinesterase Inhibition Assays

Cholinesterase enzymes including AChE derived from *Electrophorus electricus* and BChE derived from equine serum were used for evaluation of *in vitro* inhibitory potential of β-sitosterol using Ellman’s assay (Classics Ellman et al., 1961). The principle of these assays is based on the hydrolytic breakdown of acetylthiocholine iodide or butyrylthiocholine iodide via AChE or BChE leading to the formation of 5-thio-2-nitrobenzoate anion which subsequently forms a complex with DTNB to give yellow color product, which is assayed via spectrophotometer beside the reaction time (Ullah et al., 2015; Ahmad et al., 2016). Following Ellman’s assay, standard drug and β-sitosterol solutions were prepared in concentrations ranging from 12.25 to 1000 μg/ml. To prepare 0.1 M phosphate buffer (8.0 ± 0.1 pH), K_2_HPO_4_ (17.4 g/L) and KH_2_PO_4_ (13.6 g/L) solutions were prepared. The final pH was adjusted using KOH solution. Enzymes solutions were prepared by dissolving AChE (518 U/mg solid) and BChE (7–16 U/mg) in phosphate buffer and diluted to final concentrations of 0.03 U/ml and 0.01 U/ml respectively. Other solutions including DTNB (0.0002273 M), ATchI and BTchI (0.0005 M) were made using distilled water and refrigerated at 8°C in tight eppendorf tubes. The positive control (galanthamine) solution was prepared in methanol in the same concentrations as that of β-sitosterol (Ali et al., 2017).

#### Spectroscopic Analysis

For spectroscopic analysis, 5 μl from each enzyme solution was added to already assigned wells of microplate with subsequent addition of the test samples (205 μl) and DTNB reagent (5 μl). The resultant mixture was stored at 30°C for 15 min and finally the substrate solution (5 μl) was added. The 96 wells microplate reader (BioTek Instruments, United States) was used to measure the absorbance at 412 nm. Galanthamine was used as positive control (10 μg/ml), whereas, negative control consist of all reaction components except the test samples. The absorbance values along with the reaction times were recorded for 4 min at 30°C. All experiments were repeated thrice. The enzymes activities and enzyme inhibition by positive control and tested samples were calculated from the rate of absorption with change in time:

$$\begin{array}{c} V=\frac{\Delta \mathrm{Abs}}{\Delta t}\;\\ \%enzyme activity=\frac{V}{\mathrm{Vmax}}\times 100\;\\ \%enzyme inhibition=100\;-\;\%\;enzyme activity \end{array}$$

(Where V denotes rate of reaction in the present of inhibitor and Vmax denotes rate of reaction without inhibitor).

## Antioxidant Studies

### 1,1-Diphenyl, 2-Picrylhydrazyl (DPPH) Free Radicals Scavenging Assay

The ability of β-sitosterol to scavenge DPPH free radicals was evaluated following the previously reported procedure (Ahmad et al., 2015; Sadiq et al., 2015). The control drug and test samples (0.1 ml) with increasing concentration of 12.5–1000 μg/ml were added to 0.004% methanolic solution of DPPH in 96 wells microplate reader. Following 30 min incubation in dark, the absorbance was measured at 517 nm. Ascorbic acid was used as positive control in the same concentrations as that of sample. The percent scavenging potentials were determined using the following formula;

$$\%\;\mathrm{Scavenging}\;\mathrm{activity}\;=\;A_{0}\;-\;A_{1}{/A}_{0}\;\times \;100$$

where, A_0_ represent absorbance of control and A_1_ is the absorbance of sample. Each experiment was performed in triplicate.

### 2,2-Azinobis [3-Ethylbenzthiazoline]-6-Sulfonic Acid (ABTS) Radicals Scavenging Assay

The scavenging effect of β-sitosterol was also evaluated using the 2,2-azinobis [3-ethylbenzthiazoline]-6-sulfonic acid (ABTS) free radicals scavenging assay (Re et al., 1999; Kamal et al., 2015). This assay measures the ABTS radical cations scavenging potentials of antioxidants at 734 nm. For this assay, solutions of ABTS (7 mM) and potassium persulphate (2.45 mM) were freshly prepared and mixed. The resultant solution was stored at room temperature in dark place for 12–16 h to obtain a dark colored solution rich with ABTS radical cations. Subsequently, ABTS solution was diluted using 0.01 M phosphate buffer (pH 7.4), and the absorbance value was adjusted to 0.70 at 734 nm. The radical scavenging activity of the samples was measured by mixing 300 μl of test sample with 3.0 ml of ABTS solution in microplate wells. The resultant solutions were mixed for one min and the reduction in the absorbance was measured continuously for six min. Ascorbic acid was used as positive control. The assay was repeated in triplicate and the percentage inhibition was calculated using the following equation:

%Scavenging effect=Control absorbance−Sample absorbanceControl absorbance×100

### Hydrogen Peroxide (H_2_O_2_) Scavenging Assay

The H_2_O_2_ scavenging activity of β-sitosterol and ascorbic acid were evaluated using a standard analytical method (Ruch, 1989). Briefly, H_2_O_2_ solution (2 mM) was prepared in 50 mM phosphate buffer at a pH of 7.4. From each dilution of test samples, 0.1 ml was taken in test tubes and adjusted to final volume of 0.4 ml using 50 mM phosphate buffer. Finally, 06 ml of H_2_O_2_ was added to the mixture tubes and thoroughly vortexed. The final absorbance was measured at 230 nm against a blank after 10 min. The H_2_O_2_ scavenging activity was calculated using the following equation:

$$\%\mathrm{Scavenging}=\frac{Absorbance of sample}{Absorbance of control}\times 100$$

## *In Vivo* Studies

### Animals

A sub-strain of double transgenic mice JAX^®^Strain 006554, MMRRC034840 B6SJL-Tg (APPSwFlLon, PSEN1^∗^M146L^∗^L286V) 6799Vas/Mm obtained from Jackson Laboratory United States were used as disease control animals group. During genotyping, the animals lacking transgene were considered as wild/normal and were used as normal control animals. Animals were mixed breed including both male and female mice. All experiments were performed according to the guidelines of the Institute of Laboratory Animal Resources, Commission on Life Sciences, National Research Council 1996 (Clark, 1896).

### Genotyping

**FIGURE 2:**
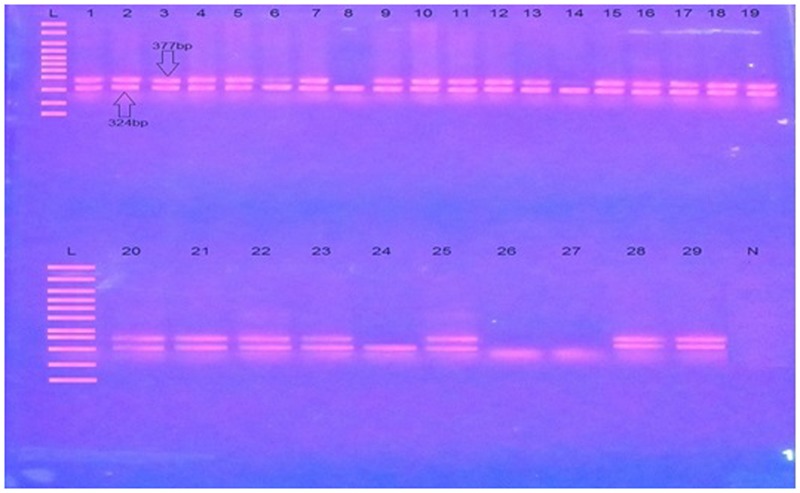
A representative image of PCR analysis of Tg APP. L is molecular weight marker (100 bp). N is negative control. 1–29 are test samples. Transgenic samples are shown by the presence of double bands one of transgene (377 bp) while other of internal positive control (324 bp), while non transgenic samples lack transgene (377 bp).

A sub-strain of transgenic mice JAX^®^Strain 006554, MMRRC034840 B6SJL-Tg (APPSwFlLon, PSEN1^∗^M146L^∗^L286V) 6799Vas/Mm, which was imported from Jackson Laboratory United States were genotyped for confirmation of generic APP transgene presence. The genotyping protocol specific for this strain was adopted with minor modifications from Jackson Laboratory United States as summarized in the **Supplementary File S2**. A representative image of PCR analysis of Tg APP and primers detail is provided in **Table 1** and **Figure 2**.

**Table 1:** Primers sequences for standard multiplex PCR reaction.

Primer	Sequence 5′→3′	Primer Type
IMR3610	AGG ACT GAC CAC TCG ACC AG	Transgene
IMR3611	CGG GGG TCT AGT TCT GAC T	Transgene
IMR7338	CTA GGC CAC AGA ATT GAA AGA TCT	Internal positive (F)
IMR7339	GTA GGT GGA AAT TCT AGC ATC ATC C	Internal positive (R)

## Behavioral Assessments

### Animal Groups

The animals were divided into four groups with each group consisting of eight animals. The first group was assigned as transgenic-galanthamine group, consisting of transgenic animals maintained on standard anti-cholinesterase drug. The second group was assigned as transgenic-saline group which consisted of transgenic animals maintained on normal saline. The third group was labeled as transgenic-β-sitosterol group, which consisted of diseased animals maintained on the test compound, β-sitosterol (10 mg/kg body weight/day for 5 consecutive days). The fourth group was non-transgenic-saline group which was consisted of wild/non-transgenic animals that were screened during genotyping. This group was maintained on normal saline and assigned as negative control. Whereas, for the *in vitro* DPPH assay, the transgenic-ascorbic acid group was used as standard drug treated group.

### Shallow Water Maze (SWM) Test

The testing method and design of paddling pool (MK2/octagonal) was adopted as previously reported from the laboratory of Deacon (2013). The apparatus walls contained eight true/false exits among which one was true, whereas, seven were false exits occluded by plastic plugs. The true exit was opened into a black plastic pipe that can be separated so that mice can be safely transferred to their respective cages. The temperature of water in the SWM apparatus was maintained at 20–25°C to provide sufficient escape motivation to the mice. The SWM was 86 cm in diameter and the exit tubes were 40 mm in diameter. The pool was placed in a test room and several clues exterior to the maze were evident from the pool (e.g., pictures, lamps, etc.), which could be used by the experimental animals for spatial orientation. The positions of the cues were constantly maintained during the experimental process. A representative structure diagram of SWM is provided as **Supplementary File S3**.

#### Training Trials

The experimental animals (both normal and transgenic) were properly trained on the test apparatus prior to drugs administration. Each animal received three trials daily (each trial one h apart) for nine consecutive days. While starting a trial, a mouse was positioned in the middle of the pool in front of one of four positions on the perimeter (9, 12, or 3 o’clock if the escape tube was at 6 o’clock). The placement was semi-random; an utmost of three successive trials were in the same direction. The animals were released when they were just above the water surface and were dropped immediately to avoid struggling behavior and to prevent impairment during initial orientation. A single trial lasts for 60 s and animals that failed to find escape way within the allocated 60 s were manually guided to the exit via glass paddles. The escape latency in seconds was recorded.

#### Test Trials

All the four groups of animals were administered with respective drugs as mentioned in the above section. After 1 h of intraperitoneal (i.p.) administration of drugs, the animals were subjected to SWM apparatus and the escape latency was recorded.

### Y-Maze Test

The Y-maze equipment consisted of three arms made of clear polystyrene or Perspex, with each arm having dimensions of 30 × 8 × 20 cm. The apparatus was mounted on a white base to improve aversiveness of the apparatus floor and to encourage escape propensity for experimental animals. During experiments a low water level (2 cm deep) was maintained for smooth paddling and easy escape of mice. The tool was provided with three true/false exits (one true and two false exits) like that of SWM. The true exit ended in a removable black pipe from which animals can be safely transferred to their respective cages.

#### Running the Paddling Y-Maze

All animals were trained for 9 days with each animal received three trials per day (each trial 1 h apart). At the start of each trial a mouse was placed at the end of one of the closed arms, facing away from the middle. The selection of arms was made with semi-random sequence of starting position and not more than 3 successive trials were done at the same position. The duration of each trial was 60 s and animals that failed to access the exit hole were manually guided to the open end of the arm via transparent Perspex slides.

#### Test Trials

The animals were divided into four groups including transgenic-galanthamine, transgenic-saline, transgenic-β-sitosterol and non-transgenic-saline group as mentioned above for the SWM test. After the training trials, transgenic-galanthamine and transgenic-β-sitosterol animals were administered galanthamine (8 mg/kg) and β-sitosterol (10 mg/kg) via i.p. route. The non-transgenic-saline and transgenic-saline groups received saline and standard diet as provided to other animals. One hour after drugs administrations, the animals were again subjected to Y-Maze task. The escape latency and spontaneous alternation behavior (SAB) of animals were recorded. At day 5, SAB was observed by placing each animal at the center of Y-Maze apparatus and freely allowed to move through the maze for three sessions, each of 8 min. The numbers of arm entries were recorded. The spontaneous alternation behavior was defined as the consecutive entry of animal into the three arms in overlapping triplet sets. The alternation behavior (%) was determined using following formula;

$$\%Alternation behavior=\left\{\frac{\begin{array}{c} Successive triplet sets\\ (entries into three different\\ arms consecutively) \end{array}}{\begin{array}{c} Total number of arm\mathrm{entries} \end{array}}\right\}-2\times 100$$

### Balance Beam Test

#### Training Trials

All animals were trained on a balance beam apparatus for five consecutive days prior to drugs administration to reduce neophobia. The animals were placed across the open space of beam which was made of large tubes/wood and lined with foam as cushioning agent to reduce injury to the animal. The start side was more brighten in comparison to the end side which was darker and ended in a dark hide. The animals were allowed to cross the beam with gentle guidance till they readily crossed. The same groups of animals were used as mentioned above, including galanthamine as standard drug.

#### Test Trials

The animals were administered respective drugs and were allowed to cross the beam. The time to cross the beam successfully was recorded.

### Evaluation of Cholinesterases in the Frontal Cortex and Hippocampus

#### Isolation of Frontal Cortex and Hippocampus

At the end of experiments, all groups of animals were sacrificed by decapitation under ether anesthesia. The FC and HC were dissected in ice cold 0.1 M phosphate buffer saline (pH 8.0). These tissues were homogenized in ice cold 0.1 M phosphate buffer saline (pH 8.0) using a homogenizer. The homogenates were centrifuged at 1000 ×*g* for 10 min at 4°C, and the supernatant was used for assay of AChE and BChE following Ellman’s procedure and previously reported protocols (Classics Ellman et al., 1961; Trevisan et al., 2003).

#### Cholinesterase Inhibition Assays of Frontal Cortex and Hippocampus

The AChE and BChE activities were measured in the FC and HC homogenates of mice brain which was standardized for protein content (5 mg/ml) as previously reported (Bradford, 1976; Isomae et al., 2002).

#### DPPH Free Radical Scavenging Assay of Frontal Cortex and Hippocampus

For assessment of antioxidant activity of post-treatment brain homogenates, the DPPH free radicals scavenging assay was used as previously reported (Braca et al., 2001). The standardized tissue homogenates (0.1 mg/ml protein) from FC and HC of all treated groups were homogenized in 1 ml methanol with subsequent addition of 0.4 ml of 0.1 mM DPPH solution. Control consisted of pure DPPH solution without any sample. The solutions were incubated at 37°C for 30 min and the absorbance was measured at 517 nm using a UV spectrophotometer (Thermo electron corporation, United States). The percentage inhibition of DPPH free radicals was calculated by using the following equation:

$$\%DPPH inhibition=\;\frac{Absorbance of control-Absorbance sample}{Absorbance of control}\times 100$$

### Molecular Docking Study

The molecular docking study was performed via MOE to predict the binding mode of β-sitosterol in the active sites of AChE and BChE as inhibitor (Rahim et al., 2016). The three dimensional structure of β-sitosterol was built using builder tool of MOE software and was protonated and energy minimized. The 3D structures of the two target enzymes (AChE and BChE) were downloaded from the protein databank (PDB) PDB ID: 1ACL and 4BOP respectively. The water molecules were removed from each protein and were 3D protonated. The energy minimization was then carried out for the stability of proteins by using the default parameters of MOE. For docking studies, the default parameters of MOE were used i.e., Placement: Triangle Matcher, Rescoring 1: London dG, Refinement: Forcefield, Rescoring 2: GBVI/WSA. For each protein, 10 different conformations of ligand were allowed to be formed and the top ranked conformations on the basis of docking score were selected for further analysis.

### Statistical Analysis

All the assays were performed in triplicate and values were expressed as mean ± SEM. In the cholinesterase and antioxidant assays (*in vitro*), one way ANOVA followed by Dunnett’s *post hoc* test was applied for comparison of positive control with the test group at 95% confidence interval. In SWM tests, two-way repeated measures ANOVA followed by *post hoc* Bonferroni’s analysis was used. In the Y-Maze and brain tissue analysis, one-way ANOVA followed by *post hoc* Tukey’s test was used. A *p*-value of <0.05 was considered as statistically significant. All statistical analyses were conducted using GraphPad Prism 5 (GraphPad Software Inc. San Diego, CA, United States).

## Results

### Structural Characterization of β-Sitosterol

The isolated compound, β-sitosterol was obtained as white-yellowish crystalline powder (2.5 g). The observed R_f_ value was 0.56 (*n*-hexane/ethyl acetate 4:1). ^1^H NMR (400 MHz, CDCl_3_) (ppm): 0.56–0.78 (m, 12H), 0.81–0.93 (m, 9H), 0.95–1.66 (m, 8+7+3H) 1.73-1.80 (m, 4H), 2.12–2.33 (m, 3H), 2.34–2.46 (m, 2H), 3.47–3.51 (m, 1H, the H-atom attached to the tertiary carbon at α-position to the OH group), 5.21–5.24 (m, 1H, represent the H-atom on the double bond). ^13^C NMR (100 MHz, CDCl_3_) (ppm): 13.91, 18.73, 18.78, 19.89, 22.02, 24.67, 25.05, 27.96, 28.36, 31.11, 31.77, 32.25, 33.85, 35.47, 36.48, 37.77, 38.99, 43.25, 45.20, 49.27, 56.96, 57.75, 63.11, 63.81, 64.36, 64.88, 65.98, 66.81, 71.26, 82.39, 101.50, 121.33, 141.29, 144.89, 148.01. The observed molecular weight by ESI-MS was 414.4. The chemical structure is shown in **Figure 1**, while the NMR and mass spectra are provided as **Supplementary File S1**.

### *In Vitro* Inhibition of Cholinesterases by β-Sitosterol

The results of *in vitro* AChE and BChE inhibitory potentials of β-sitosterol are given in **Table 2**. A concentration dependent inhibitory activity was shown by β-sitosterol against AChE and BChE. The percent inhibition was observed as 43 and 39.50% at a concentration of 31.25 μg/ml, while it was 83.5 and 89.16% at a concentration of 1000 μg/ml with an IC_50_ of 55 μg/ml and 50 μg/ml against AChE and BChE, respectively. Similarly, the percent inhibition afforded by the positive control, galanthamine against AChE and BChE was 51.66 and 42.66% at a concentration of 31.25 μg/ml, while at 1000 μg/ml it was observed as 91.10 and 94.16% having an IC_50_ value of 10 μg/ml and 8 μg/ml, respectively. The inhibition produced by β-sitosterol against AChE was significant at concentrations of 62.5–250 μg/ml (*P* < 0.05) and 500 (*P* < 0.01), compared to that produced by galanthamine.

**Table 2:** Results of *in vitro* cholinesterase inhibitory potential of β-sitosterol.

Samples	Concentration μg/ml	Acetylecholinesterase		Butyrylcholinesterase	
		% inhibitions	IC_50_ μg/ml	% inhibitions	IC_50_ μg/ml
β-sitosterol	31.25 62.50 125 250 500 1000	43.00 ± 2.64^ns^ 52.33 ± 1.76^∗^ 59.00 ± 2.30^∗^ 67.66 ± 2.70^∗^ 75.33 ± 2.02^∗∗^ 83.50 ± 2.46^ns^	55	39.50 ± 1.89^ns^ 54.33 ± 1.76^ns^ 65.00 ± 2.30^ns^ 71.50 ± 4.72^ns^ 78.00 ± 1.73^ns^ 89.16 ± 1.58^ns^	50
Galanthamine	31.25 62.50 125 250 500 1000	51.66 ± 1.76 65.33 ± 2.90 70.83 ± 2.60 79.00 ± 2.58 86.83 ± 1.87 91.10 ± 1.42	10	42.66 ± 2.60 60.00 ± 3.21 71.16 ± 1.92 79.66 ± 1.76 85.33 ± 2.16 94.16 ± 2.40	8

*Values expressed as means ± SEM. A *P*-value of less than 0.05 was considered as statistically significant. Values significantly different when compared to standard drug (galanthamine) at the same concentration, i.e., ^∗^*p* < 0.05 and ^∗∗^*p* < 0.01 ns: Values not significantly different when compared to standard drug (*p* > 0.05).*

### *In Vitro* DPPH Radicals Scavenging Activity of β-Sitosterol

The *in vitro* DPPH free radical scavenging assay revealed antioxidant activity of β-sitosterol. As shown in **Table 3**, a concentration dependent scavenging effect against DPPH free radicals was showed by β-sitosterol, which implies its concentration dependent antioxidant activity. A low percent inhibition was observed as 11% at a concentration of 12.5 μg/ml, while high inhibition was observed at a maximum concentration of 1000 μg/ml. Similarly, ascorbic acid which used as positive control also produced a concentration dependent inhibitory effect (51% at 12.5 μg/ml and 89.50% at 1000 μg/ml). The IC_50_ for the DPPH free radical scavenging property of β-sitosterol and ascorbic acid were calculated as 140 μg/ml and 50 μg/ml, respectively. The percent inhibition of β-sitosterol was significantly different from that of ascorbic acid at a concentration of 12.5–200 μg/ml (*P* < 0.001) and 400–800 (*P* < 0.05).

**Table 3:** Antioxidant effect of β-sitosterol in the DPPH, ABTS and H_2_O_2_ radicals scavenging assays.

Samples	DPPH free radical Scavenging			ABTS free radical scavenging		H_2_O_2_ free radical scavenging	
	Conc. μg/ml	% Scavenging activity	C_50_ μg/ml	% Scavenging activity	IC_50_ μg/ml	% Scavenging activity	IC_50_ μg/ml
β-sitosterol	12.5	11.00 ± 1.15^∗∗∗^	140	16.83 ± 1.58^∗∗∗^	120	9.00 ± 1.15^∗∗∗^	280
	25	16.16 ± 1.30^∗∗∗^		22.50 ± 1.04^∗∗∗^		14.66 ± 1.76^∗∗∗^	
	50	21.33 ± 2.02^∗∗∗^		30.16 ± 2.16^∗∗∗^		23.00 ± 0.57^∗∗∗^	
	100	38.33 ± 1.76^∗∗∗^		36.83 ± 1.09^∗∗∗^		34.66 ± 0.98^∗∗∗^	
	200	51.00 ± 2.30^∗∗∗^		43.91 ± 1.62^∗∗∗^		49.33 ± 0.72^∗^	
	400	65.00 ± 0.57^∗^		69.83 ± 1.09^∗^		56.83 ± 1.30^∗∗^	
	800	71.66 ± 3.17^∗^		76.00 ± 3.46^∗^		64.00 ± 1.15^∗∗^	
	1000	79.66 ± 1.76^ns^		84.66 ± 2.90^ns^		71.66 ± 2.59^∗∗^	
Positive control	12.5	32.50 ± 1.22	50	49.00 ± 0.57	20	31.00 ± 0.57	65
	25	43.50 ± 2.04		60.00 ± 0.94		40.00 ± 1.15	
	50	50.00 ± 1.00		68.83 ± 1.01		47.33 ± 1.20	
	100	66.75 ± 4.69		72.66 ± 2.02		52.16 ± 0.49	
	200	70.50 ± 0.40		78.66 ± 0.72		60.66 ± 1.20	
	400	76.00 ± 1.22		81.83 ± 1.64		73.00 ± 1.52	
	800	83.75 ± 0.61		88.00 ± 0.57		80.00 ± 0.47	
	1000	89.50 ± 2.04		92.66 ± 2.51		85.00 ± 0.94	

*Values expressed as means ± SEM. A *P*-value of less than 0.05 was considered as statistically significant. Values significantly different when compared to standard drug (ascorbic acid) at the same concentration, i.e., ^∗^*p* < 0.05, ^∗∗^*p* < 0.01, ^∗∗∗^*p* < 0.001 and ns: Values not significantly different when compared to standard drug.*

### *In Vitro* ABTS Radicals Scavenging Activity of β-Sitosterol

The ABTS anti-radical assay also showed free radical scavenging property of β-sitosterol. As shown in **Table 3**, with increasing β-sitosterol concentration, there is an increased percent scavenging of ABTS radicals. This concentration dependent free radical scavenging effect was observed as 16.83 and 84.66% at lower and higher concentrations of 12.5 μg/ml and 1000 μg/ml, respectively. Similar trend in the percent inhibition was also produced by the positive control, ascorbic acid which showed an inhibition of 49% at 12.5 μg/ml and 92.66% at 1000 μg/ml. The percent inhibition of β-sitosterol was significantly different from that of ascorbic acid at 12.5–200 μg/ml (*P* < 0.001) and 400–800 μg/ml (*P* < 0.05). The IC_50_ value for ABTS anti-radical activity of β-sitosterol was 120 μg/ml, while it was 20 μg/ml for ascorbic acid.

### *In Vitro* H_2_O_2_ Radicals Scavenging Activity of β-Sitosterol

The H_2_O_2_ radicals scavenging assay further corroborated the anti-radical property of β-sitosterol. As shown in **Table 3**, with an increase in the concentration of β-sitosterol, a linear increase in the scavenging of H_2_O_2_ radicals was observed. At a lower concentration of 12.5 μg/ml, the percent scavenging by β-sitosterol was 9%; however, at a higher β-sitosterol concentration (1000 μg/ml), the maximum inhibition was noted as 71.66%. Likewise, ascorbic acid which was as positive control also produced a concentration dependant percent inhibition of H_2_O_2_ radicals, i.e., 31% at a lowest concentration (12.5 μg/ml) and 85% at a highest concentration (1000 μg/ml). A significant difference in the percent scavenging effect was observed at concentrations of 12.5–100 μg/ml (*P* < 0.001), 200 μg/ml (*P* < 0.05) and 400–1000 μg/ml (*P* < 0.01) of β-sitosterol, compared to respective concentrations of ascorbic acid. The concentration at which β-sitosterol produced 50% inhibition of H_2_O_2_ free radicals was observed as 280 μg/ml, while it was 65 μg/ml for ascorbic acid.

### Effect of β-Sitosterol in the Shallow Water Maze Test

**FIGURE 3:**
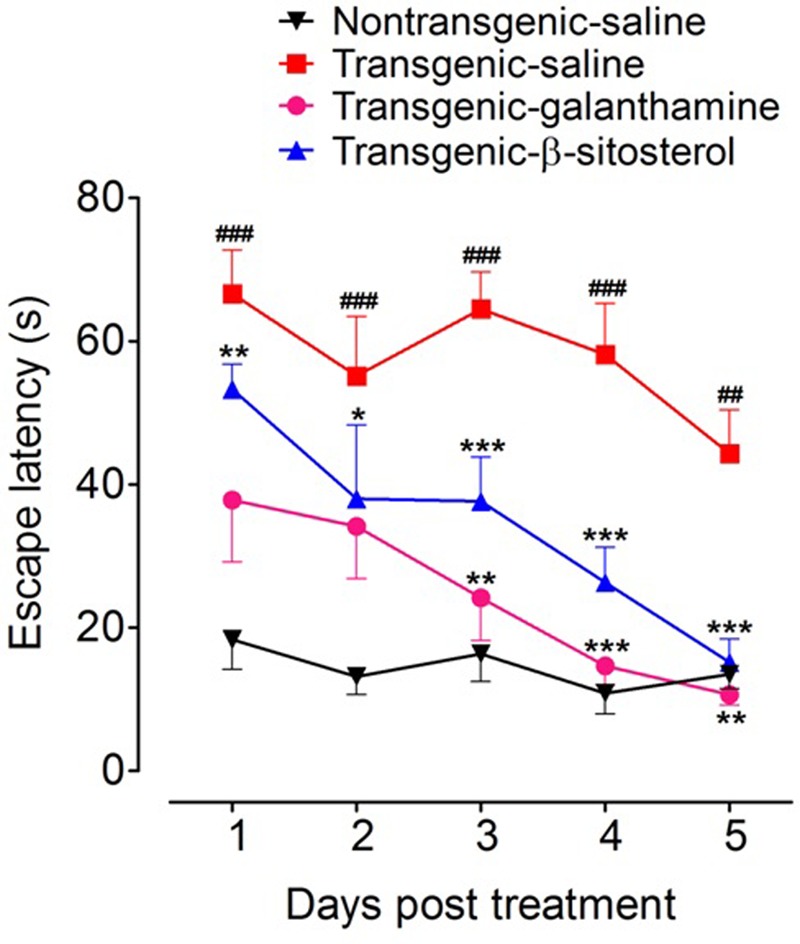
Results of shallow water maze (SWM) test in different groups of animals; Non-transgenic-saline, Transgenic-saline, Transgenic-β-sitosterol and Transgenic-galanthamine. Symbols represent mean escape latency in s ± SEM. ^##^*P* < 0.01, ^###^*P* < 0.001 compared to non-transgenic normal controls, ^∗^*P* < 0.05, ^∗∗^*P* < 0.01, ^∗∗∗^*P* < 0.001 compared to transgenic saline treated controls, two-way repeated measures ANOVA followed by *post hoc* Bonferroni’s analysis.

As shown in **Figure 3**, treatment with galanthamine and β-sitosterol produced significant changes in the escape latency [time = (*F*_4,75_ = 9.40, *P* < 0.0001), treatment = (*F*_3,75_ = 54.92, *P* < 0.0001), interaction = (*F*_12,75_ = 1.29, *P* = 0.2435)]. The transgenic animals showed significant increase (*P* < 0.001 during days 1–4 and *P* < 0.01 after day 5) in the flight latency compared to non-transgenic normal controls. Treatment with β-sitosterol shortened the escape duration as the transgenicity induced maze durance was significantly decreased during post treatment day 1 (*P* < 0.01), day 2 (*P* < 0.05) and after 3–5 days (*P* < 0.001). Likewise, the positive control, galanthamine also showed beneficial effect as it keep normalizing the transgenicity induced prolonged escape throughout the time-period, but a significant effect was only noted after day 3 (*P* < 0.01), day 4 (*P* < 0.001) and day 5 (*P* < 0.01) when compared to transgenic saline treated animals.

### Effect of β-Sitosterol in the Y-Maze Test

**FIGURE 4:**
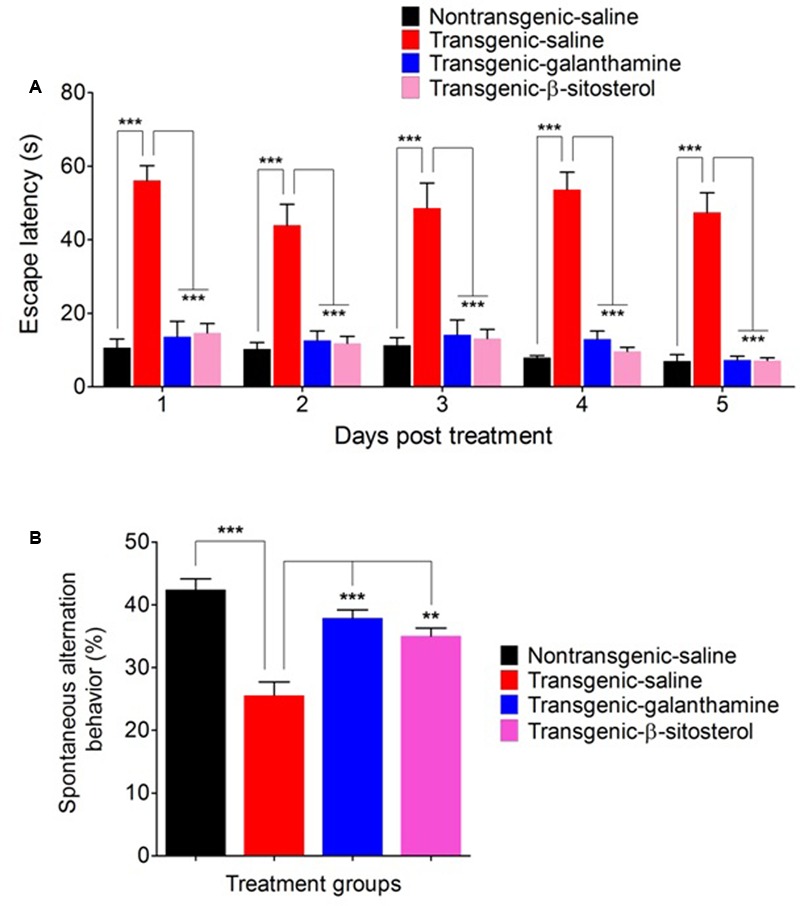
Effect of galanthamine and β-sitosterol on escape latency **(A)** and percent spontaneous alternation behavior **(B)** in the Y maze test in different groups of animals; Non-transgenic-saline, Transgenic-saline, Transgenic-β-sitosterol and Transgenic-galanthamine. Bars represent mean ± SEM. ^∗∗^*P* < 0.01, ^∗∗∗^*P* < 0.001, two-way repeated measures ANOVA followed by *post hoc* Bonferroni’s analysis or one-way ANOVA followed by *post hoc* Tukey’s multiple comparison test.

**Figure 4** shows a significant effect of treatment with galanthamine and β-sitosterol on the escape latencies [time = (*F*_4,75_ = 3.57, *P* = 0.0194), treatment = (*F*_3,75_ = 151.10, *P* < 0.0001), interaction = (*F*_12,75_ = 0.52, *P* = 0.8933)] and percent spontaneous alternation behavior (*F*_3,24_ = 18.50, *P* < 0.0001) of animals in the Y maze test paradigm. The transgenic saline treated animals displayed a prolonged stay in the Y maze as a significant increase (*P* < 0.001) in the escape behavior was observed throughout the study time-period (days 1–5), compared to the non-transgenic saline treated controls. However, treatment with either β-sitosterol or the positive control, galanthamine attenuated the prolongation of maze durance as these treated transgenic animals exhibited a significant decrease (*P* < 0.001) in the escape latency throughout the treatment days when compared to the transgenic saline treated animals (**Figure 4A**). The transgenic saline treated mice strain showed a significant decrease (*P* < 0.001) in the percent spontaneous alternation as compared to the non-transgenic saline treated normal controls. However, this decrement in the percent alternation was significantly reversed by treatment with either galanthamine or β-sitosterol, thus showing an improvement in spatial memory (**Figure 4B**).

### Effect of β-Sitosterol in the Balance Beam Test

**FIGURE 5:**
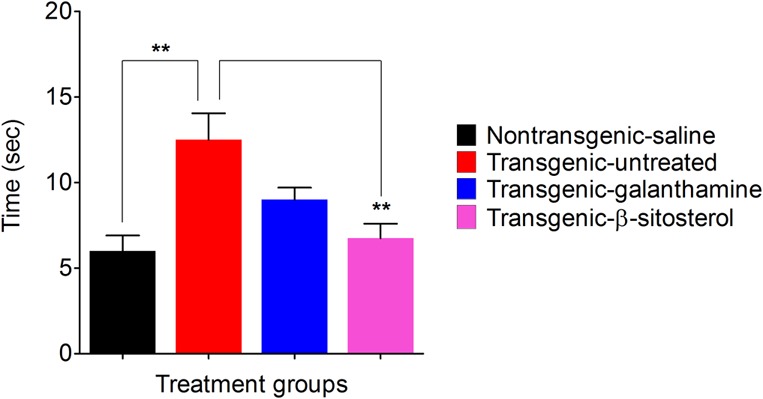
Effect of β-sitosterol on motor coordination and balance in the balance beam test.

As shown in **Figure 5**, the non-transgenic-saline, transgenic-saline and transgenic-galanthamine treated animals reached the allocated target in 6.33 ± 1.20, 11.00 ± 0.57, and 6.66 ± 1.20 min, respectively. In comparison, the transgenic-β-sitosterol group reached the target in 9.33 ± 0.88 min.

### Effect of β-Sitosterol on Cortical and Hippocampus AChE, BChE Activities

**FIGURE 6:**
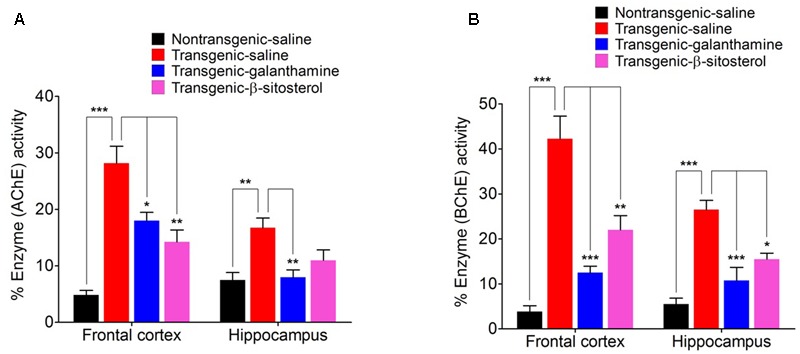
Effect of β-sitosterol on the activities of AChE **(A)** and BChE **(B)** in the frontal cortex and hippocampus of different groups animals. Bars represent mean percent AChE or BChE activity ± SEM. **^∗^***P* < 0.05, **^∗∗^***P* < 0.01, ^∗∗∗^*P* < 0.001, one-way ANOVA followed by *post hoc* Tukey’s test.

As shown in **Figures 6A,B**, galanthamine and β-stisterol produced significant changes in the percent enzyme activities of AChE and BChE in the FC (*F*_3,12_ = 22.77, *P* < 0.0001 and *F*_3,12_ = 27.75, *P* < 0.0001), and HC (*F*_3,12_ = 7.488, *P* = 0.0044 and *F*_3,12_ = 19.62, *P* < 0.0001), respectively. The transgenic animals were associated with significant increase in the enzymatic activities of AChE (*P* < 0.001, *P* < 0.01) and BChE (*P* < 0.001) in both the FC and HC brain regions. In the FC, significant decrease in AChE and BChE was observed with galanthamine (*P* < 0.05, *P* < 0.001) and β-sitosterol (*P* < 0.05, *P* < 0.001). Similarly, the levels of these enzymes also significantly decreased by galanthamine (*P* < 0.01, *P* < 0.001) in the brain area of HC; however, β-sitosterol was only able to decrease the level of BChE (*P* < 0.05) in this area, compared to saline treated transgenic animals. A significant increase in the percent AChE activity (*P* < 0.01, *P* < 0.05 only in FC) was produced by galanthamine and β-sitosterol compared to non-transgenic animals. The transgenic animals treated with β-sitosterol significantly changed the activity of BChE in both the FC (*P* < 0.01) and HC (*P* < 0.05) when compared to transgenic and non-transgenic saline treated controls.

### Effect of β-Sitosterol on Cortical and Hippocampus DPPH Scavenging Activity

**FIGURE 7:**
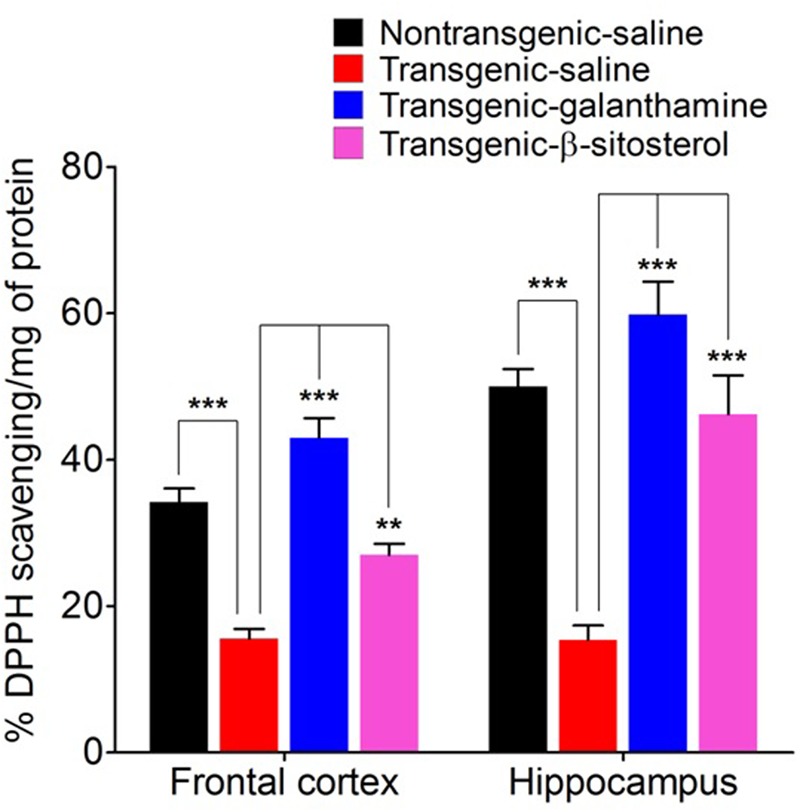
DPPH free radical scavenging activity of proteins expressed in the frontal cortex and hippocampus after treatment with galanthamine and β-sitosterol. Bars represent mean percent DPPH activity ± SEM. ^∗∗^*P* < 0.01, ^∗∗∗^*P* < 0.001, one-way ANOVA followed by *post hoc* Tukey’s multiple comparison test.

**Figure 7** shows the antioxidant status of proteins expressed in the FC (*F*_3,16_ = 36.55, *P* < 0.0001) and HC (*F*_3,16_ = 25.26, *P* < 0.0001) as measured by the *in vitro* DPPH free radical scavenging activity. Compared to the saline treated normal controls, the homogenates from the transgenic saline treated animals exhibited a significant decrease (*P* < 0.001, *P* < 0.01) in the percent scavenging activity in both the FC and HC, therefore showing a tendency of oxidative stress in these critical areas of spatial memory. Treatment with galanthamine and β-sitosterol revealed a protective effect in the transgenic strain as they significantly increased (*P* < 0.001) the scavenging of free radicals in the FC and HC brain areas and thus declined the oxidative stress which may plausibly involved in the decline of spatial memory.

### *In Silico* Binding of β-Sitosterol with AChE and BChE Active Sites

**FIGURE 8:**
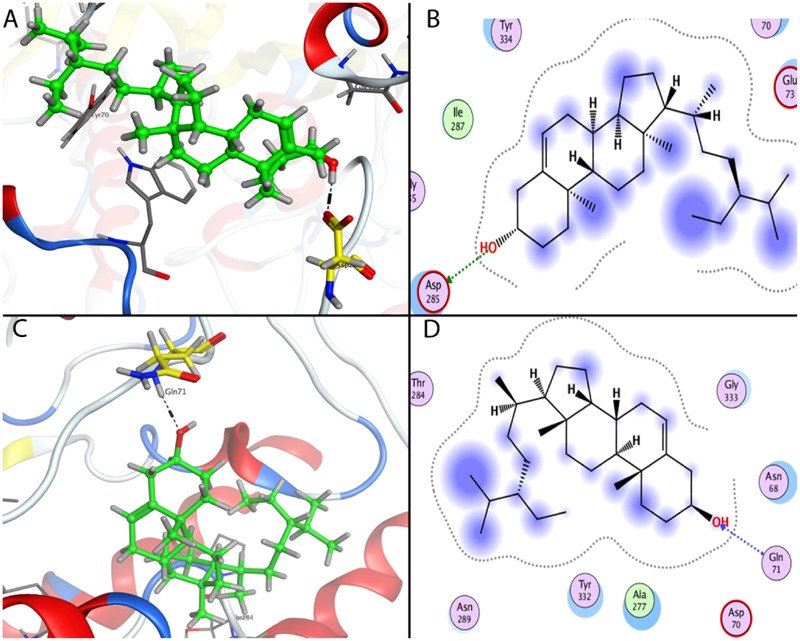
Docking conformations of β-sitosterol on acetylcholinesterase (AChE) and butyrylcholinesterase (BChE). **(A)** 3D binding mode of β-sitosterol as inhibitor of AChE. **(B)** 2D binding mode of beta-sitosterol as inhibitor of AChE showing H-donor interaction. **(C)** 3D binding mode of β-sitosterol in the binding cavity of BChE. **(D)** 2D binding mode of β-sitosterol as inhibitor of BChE showing H-acceptor interaction.

The most favorable docking poses were observed inside the binding pockets of the two proteins with proper orientation in term of binding affinity (-5.62 Kcal/mol in AChE and -5.77 Kcal/mol in BChE), solvation energy (-28.58 Kcal/mol in AChE and -30.64 Kcal/mol in BChE) and docking score -5.3168602 Kcal/mol (AChE) and -6.75507879 Kcal/mol (BChE). Such lower values indicate good fitness of the compound in the binding pocket of the protein and stable β-sitosterol-protein interaction. The analysis of the binding mode of the most favorite AChE docked conformation exposed that the compound interacted over the binding cavity with an acidic amino acid residue Asp 285 through OH group by forming hydrogen bond with carbonyl oxygen (**Figures 8A,B**) with a bond length of 2.84 Å and bond energy of -3.2 Kcal/mol. In case of BChE, the docked conformation also showed a similar interaction with NH group of Gln 71 as depicted in **Figures 8C,D**. Here bond length observed was 3.05 Å and bond energy -2.1 Kcal/mol.

## Discussion

The current study is based on previously published studies from the same laboratory, in which *P. hydropiper* crude extracts (Ayaz et al., 2014a) and essential oils (Ayaz et al., 2015) were screened against two important drug targets of AD. The crude extracts and essential oils exhibited considerable *in vitro* inhibitory activities against AChE, BChE enzymes and DPPH, ABTS and H_2_O_2_ free radicals. Based on these previous results, the most potent fractions were subjected to column chromatography to isolate β-sitosterol which was subsequently evaluated in various *in vitro* and *in vivo* studies for its potential therapeutic effectiveness in the management of AD. Cognitive decline is a major pathological complication in various neurodegenerative diseases including AD. Learning and memory are the critical parts of cognitive system. The CCS plays a vital role in the attainment of cognitive functions. It become a well-known phenomenon in the early sixties that cognitive functions are dependent on central cholinergic neurotransmission (Bartus et al., 1985; Holttum and Gershon, 1992). Although other neurotransmitters are also involved in the acquisition of cognitive functions, still the cholinergic system is of greater interest in the neurological research owing to its role in learning and memory (Hagan and Morris, 1988). In this regard, the involvement of cholinergic synapse in the storage and salvage of new information has been established (Deutsch, 1972). It is observed that the deterioration of cholinergic neurons has been attributed to the cognitive impairment experienced in AD patients (Schliebs and Arendt, 2011) that is further confirmed from the antagonistic effect of scopolamine on cholinergic system which produce deficits in attainment, retention, consolidation and recovery of memory (Stevens, 1981; Deiana et al., 2011). Thus, increasing the cholinergic tone can revert the cognitive dysfunction (Dumas and Newhouse, 2011; Haense et al., 2012; Anand et al., 2014). Several approaches have been adopted to balance the cholinergic insufficiency including the use of ACh precursors, agonists of muscarinic and nicotinic receptors; however, none of these show any efficacy due to safety, bioavailability and selectivity concerns (Fisher, 2000; Mangialasche et al., 2010). Nonetheless, inhibitors of cholinesterases (AChE, BChE) have shown promise and compounds like donepezil, galantamine, rivastigmine and tacrine have been clinically approved for conditions associated with cognitive decline (Hasselmo, 2006; Takada-Takatori et al., 2006). Subject to the efficacy and cost of these cholinesterase inhibitors, there is a dire need to search and develop more efficient and cost-effective cholinesterase inhibitors particularly from natural products.

In the current study, β-sitosterol exhibited strong anticholinesterase properties both *in vitro* and *in vivo* as well as correct the behavioral deficits, the effects of which were comparable to that produced by the standard drug. A major problem in the development of anti-AD drugs is in-sufficient bioavailability of the test compounds at the target site. The prospective beneficial effects afforded by β-sitosterol revealed that it can readily penetrate the blood brain barrier and sufficiently concentrate in the brain tissues involved in cognition and thereby inhibit esterase-mediated degradation of ACh and hence attenuate the deficit in memory and behavior.

Free radicals are implicated in a variety of disorders like neurodegeneration, ischemic heart diseases, atherosclerosis, gastritis and reperfusion injury of several tissues (Kumpulainen and Salonen, 1999; Shah et al., 2013). During oxidative metabolic processes, free radicals are generated which are subsequently transformed to non-radical forms by various enzymes like hydroperoxidases. However, during excessive radicals generation or depletion of human immune system, there is a need of exogenous radicals scavengers (Halliwell, 1994). In patients with AD, dysfunctional mitochondria liberate excessive amount of oxidizing free radicals which lead to oxidative stress, with subsequent oxidative stress related damage and pathological transformations. Amyloid (Aβ) is known as a potent originator of oxygen and nitrogen free radicals and pathologically affect neural, microglial and cerebrovascular cells (Querfurth and LaFerla, 2010). Clinically available synthetic antioxidants, like gallic acid esters, tertiary butylated hydroquinon and butylated hydroxy toluene (BHT) are associated with severe health hazards (Barlow, 1990). Several compounds from natural sources are reported to have strong antioxidant potentials and can be used as safe free radicals scavengers (Ahmad et al., 2015; Ayaz et al., 2017). In the current study, β-sitosterol showed strong antioxidant potential by scavenging free radicals of diverse nature both *in vitro* and *in vivo*.

**FIGURE 9:**
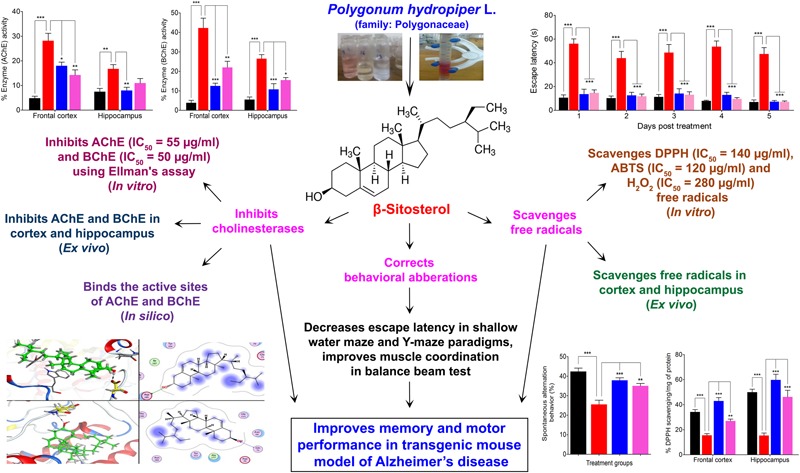
Scheme summarizing the anti-Alzheimer property of β-sitosterol isolated from *Polygonum hydropiper* L.

The anticholinesterase effect produced by β-sitosterol can be further confirmed from the *in silico* studies in which β-sitosterol produced inhibition by strongly binding to the active sites of AChE and BChE. The development of multi-potent molecules is of great interest to the scientific community, owing to their effectiveness on multiple targets of the disease and pharmacoeconomic aspects. In addition to cholinesterase inhibitions, free radicals scavenging potentials of β-sitosterol may be useful in the management of AD and other neurological disorders. Besides correction of behavioral aberrations in various memory tests, β-sitosterol also improves motor coordination and balance in transgenic animals which signify its usefulness in enhancing motor performance alongside other beneficial effects in AD patients. Muscarinic ACh receptors activation is known to enhance GABAergic mediated neurotransmission in the cortical, pyramidal neurons and play important role in the flow of information and acquisition of certain types of memory (Constantinidis et al., 2002; Zhong et al., 2003). Cholinergic hypofunction is a well known pathological aspect of AD. Our study indicated that β-sitosterol possess significant *in vitro* cholinesterase inhibitory and free radicals scavenging potentials. Additionally, β-sitosterol demonstrated AChE, BChE inhibitory activities in the FC and HC tissues of transgenic animals, which strongly suggest its ability to inhibit these target enzymes in the brain regions. Thus the level of ACh is maintained for prolong time in synaptic cleft and stimulate the cholinergic receptors. The enhanced cholinergic transmission by β-sitosterol is likely to offer useful effects for the restoration of memory in AD and in the prevention of free radicals induced neurodegeneration in aging brain (**Figure 9**).

## Conclusion

In the current study, the potential effectiveness of β-sitosterol was assessed against several pathological targets of AD. *In vitro* studies revealed that β-sitosterol possesses strong anti-cholinesterase and antioxidant potentials. Though the potency of this compound was less in comparison to standard drugs, yet its dual efficiency (enzymes inhibition and free radicals scavenging capacity) may be of potential benefit. SWM, Y-maze and balance beam studies concluded that the use of β-sitosterol can improve cognitive deficits, short term memory and locomotor disabilities. *In vivo* studies revealed that β-sitosterol adequately reach the brain and inhibit enzymes involved in the metabolism of cholinesterases and act as free radicals scavenger. *In vitro, in vivo*, behavioral and docking studies support the potential use of β-sitosterol as AD modifying agent. Further studies regarding its effects on amyloid deposition via immunohistochemistry is in progress and will provide better understanding of its therapeutic efficacy.

## Availability of Supporting Data

The data presented in this manuscript belong to research work (Thesis) of Muhammad Ayaz and has been deposited to the repository of Higher Education Commission (HEC) of Pakistan (www.hec.gov.pk). The data is not published anywhere yet. However, the materials are available to the researchers upon request.

## Ethics Statement

The study protocol was approved by Departmental Research Ethics Committee (DREC), Department of Pharmacy, University of Malakand via reference no. DREC/20160502/01. All experiments were performed according to the rulings of the Institute of Laboratory Animal Resources, Commission on Life Sciences, National Research Council (1996). Double transgenic mice (sub-strain) name [006554, MMRRC034840 B6SJL-Tg(APPSwFlLon, PSEN1^∗^M146L^∗^L286V)6799Vas/Mm] were obtained from Jackson Laboratory United States.

## Author Contributions

MA conceived the project, carried out experimental work, data collection, evaluation, literature search and manuscript preparation. MJ and FU supervised research work, helped in study design and drafted the final version of the manuscript. FS, AS, GA, MS, NA, and AA participated in project design, executed behavioral tasks and performed *in vivo* and *in vitro* studies. MS also helped in drafting the final version of manuscript. MO performed genotyping studies in collaboration with MA and GA. MES performed spectral analysis and interpretations. AW performed molecular docking studies. SA participated in “*in vitro*” and “*ex vivo*” cholinesterase assays. All authors read and approved the final manuscript for publication.

## Conflict of Interest Statement

The authors declare that the research was conducted in the absence of any commercial or financial relationships that could be construed as a potential conflict of interest.

## Supplementary Material

FILE S1 It contain NMR and Mass spectrum of β-sitosterol.

FILE S2 It contain detail process of genotyping transgenic animals.

FILE S3 It contain Shallow water maze (SWM) apparatus is provided as additional/Supplementary File.

## Abbreviations

Aβ: amyloid beta protein
ABTS: 2, 2-azinobis [3-ethylbenzthiazoline]-6-sulfonic acid
ACh: acetylcholine
AChE: acetylcholinesterase
AD: Alzheimer’s disease
APP: amyloid precursor protein
ATchI: acetylthiocholine
BACE-1: beta amyloid cleaving enzyme
BChE: butyrylcholinesterase
BTchI: butyrylthiocholine
CCS: Central cholinergic system
DPPH: 1,1-diphenyl, 2-picrylhydrazyl
DREC: Departmental Research Ethics Committee
DTNB: 5,5-dithio-bis-nitro-benzoic acid
FC: frontal cortex
H_2_O_2_: hydrogen peroxide
HC: hippocampus
IVIVC: *in vitro-in vivo* correlation
MAO: monoamine oxidase inhibitors
MOE: Molecular Operating Environment
MWM: Morris water maze
PDB: protein databank
SAB: spontaneous alteration behavior
SWM: shallow water maze
