# Supplementary figures and images for: Anti-Alzheimer’s Studies on β-Sitosterol Isolated from Polygonum hydropiper L
Source: Front Pharmacol. 2017 Oct 6;8:697. doi: 10.3389/fphar.2017.00697 (PMC5635809; doi:10.3389/fphar.2017.00697)

Supplementary File 1:

**1H NMR of β-sitosterol**


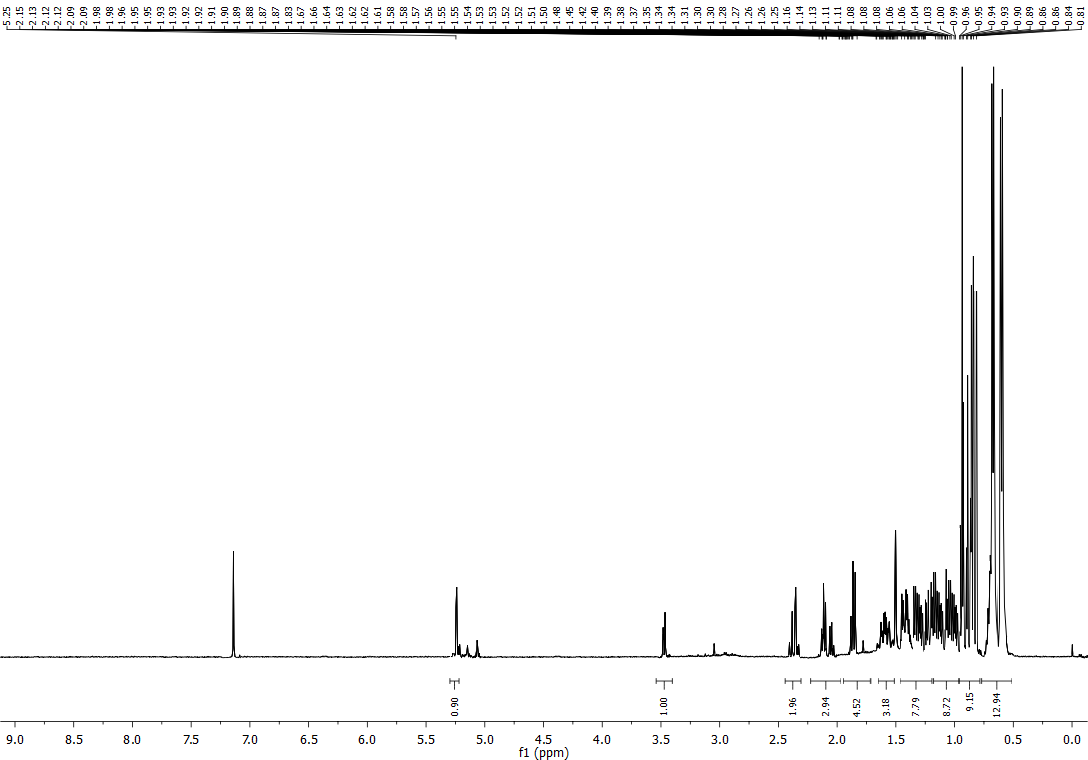


**13C NMR of β-sitosterol**

**
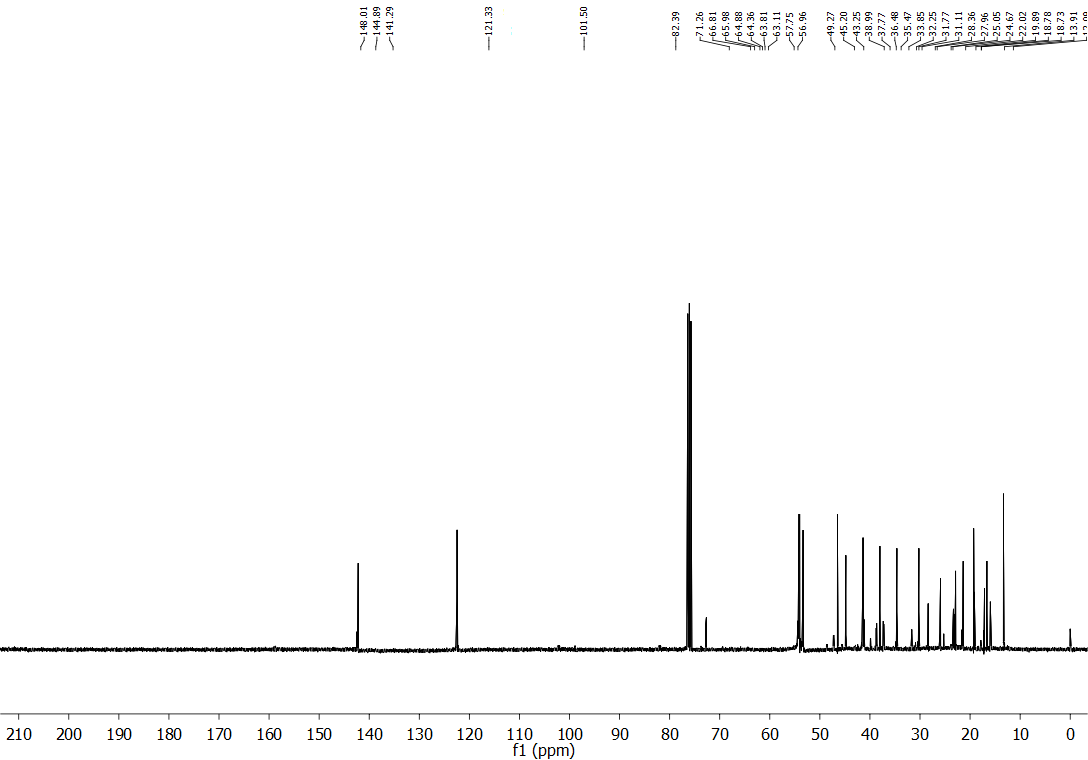
**

**MS of β-sitosterol**

**
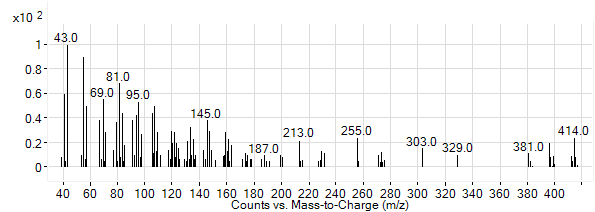
**

Supplement: FILE S1 — It contain NMR and Mass spectrum of β-sitosterol. [file Data_Sheet_1.DOCX]

**Supplementary file S3A:** Construction of Shallow water maze (SWM) and Y-Maze.


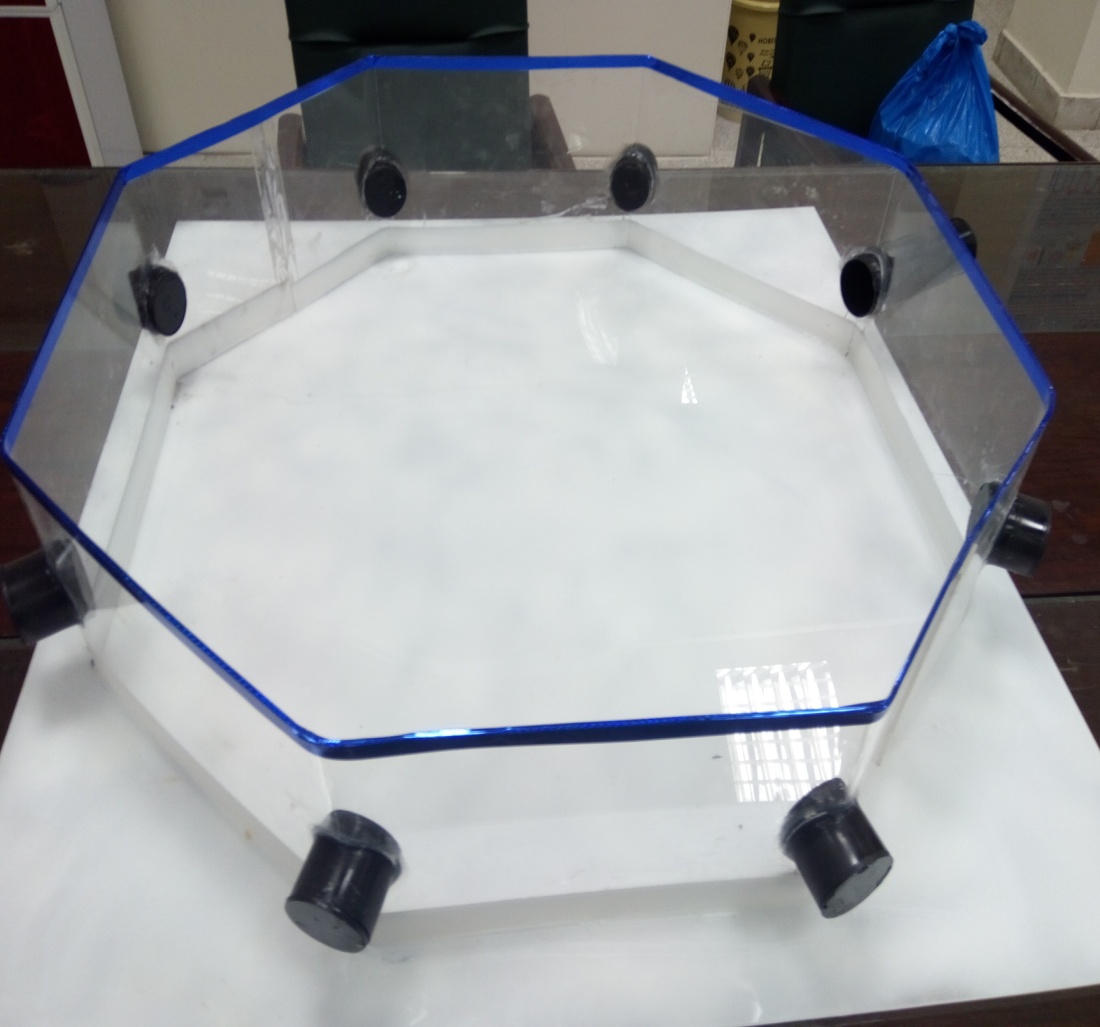


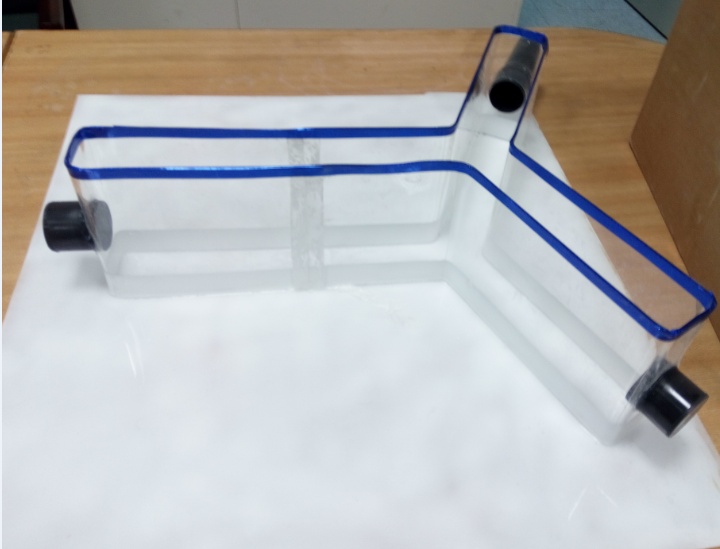

Supplement: FILE S3 — It contain Shallow water maze (SWM) apparatus is provided as additional/Supplementary File. [file Data_Sheet_3.DOCX]
