# Supplementary material for: Anti-Alzheimer’s Studies on β-Sitosterol Isolated from Polygonum hydropiper L
Source: Front Pharmacol. 2017 Oct 6;8:697. doi: 10.3389/fphar.2017.00697 (PMC5635809; doi:10.3389/fphar.2017.00697)
Supplement: FILE S2 — It contain detail process of genotyping transgenic animals. [file Data_Sheet_2.DOCX]

**Genotyping Transgenic mice strain “B6SJl-Tg”**

Transgenic mice strain “B6SJl-Tg” imported form Jackson Lab U.S was genotyped for conformation of generic APP transgene presence.

**DNA extraction and quantification**

Genomic DNA was extracted from tail tissues of 52 transgenic mice via GF-1 Tissue DNA Extraction kit (Vivantis) (Cat#GF-TD-100). Approximately 20 ug of pure genomic DNA was extracted by following the detailed protocol as per kit manual. Quantification of genomic DNA was performed via yield gel electrophoresis method, the detail of which is given in Supplementary Material I.

**Standard Multiplex PCR and Gel Electrophoresis**

DNA amplification was performed following Standard Multiplex PCR Technique. Tg APP Transgene (377bp) and Internal positive control (324bp) were amplified in one reaction using two sets of sequence specific primers (Table 1). Primer sequences for Transgene and Internal positive control “B6SJl-Tg” strain were obtained from Jackson Lab website <https://www.jax.org/strain/006554>. Optimized PCR cycling condition are mentioned in Table 2 while Master Mix components with their respective concentrations optimized for Tg APP transgene amplification are enlisted in Table 3. The detailed method for gel electrophoresis is given in Supplementary Material II.

**Table 1:** Primers sequences for Standard Multiplex PCR reaction.

| Primer Sequence 5’→3’ Primer Type |
| --- |
| IMR3610 AGG ACT GAC CAC TCG ACC AG Transgene  IMR3611 CGG GGG TCT AGT TCT GAC T Transgene  IMR7338 CTA GGC CAC AGA ATT GAA AGA TCT Internal Positive (F)  IMR7339 GTA GGT GGA AAT TCT AGC ATC ATC C Internal Positive (R) |

**Table 2:** Cycling conditions for PCR reaction.

| **Step Temperature (^o^C) Time Note** |
| --- |
| 1 94 3 min ---  2 94 30 sec ---  3 55 1 min ---  4 72 1 min Repeat step 2-4 for 35 cycles  5 72 2 min ---  6 10 --- hold |

**Table 3:** Master Mix components of PCR reaction for amplification of Tg APP Transgene.

| **Reaction Components Volume (**μl) **Final Concentration** |
| --- |
| ddH2O 3.12 -------  10X Taq Buffer 1.20 2.50  25Mm MgCl2 1.20 2.5  2.5mM dNTP 0.96 0.2  20 μM IMR 3610 (Forward Primer) 0.60 1  20 μM IMR 3611 (Reverse Primer) 0.60 1  20 μM IMR 7338 (Forward Primer) 0.30 0.5  20 μM IMR 7339 (Reverse Primer) 0.30 0.5  5U/μ Taq DNA Polymerase 0.06 0.03  DNA (20 ug/ 200μl) 3.66 0.22 ug/ μl |
| **Total Volume** 12 μl |


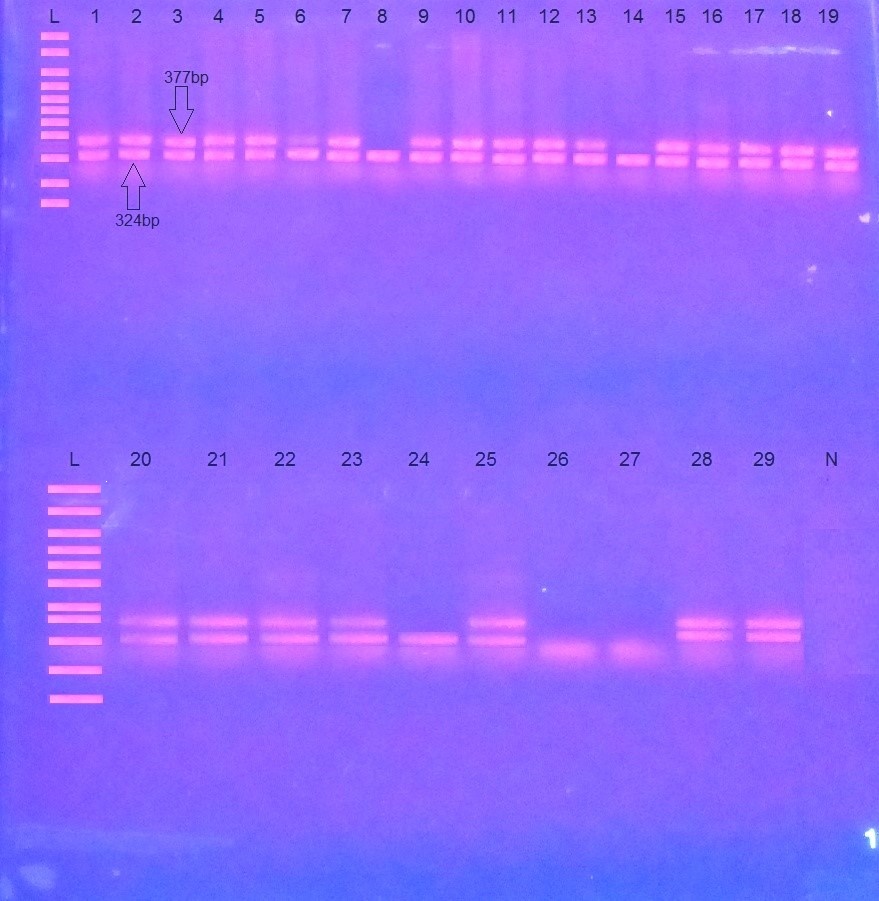


Figure 1. A Representative image of PCR analysis of Tg APP. L is molecular weight marker (100 bp). N is negative control. 1 to 29 are test samples. Transgenic samples are shown by the presence of double bands one of transgene (377 bp) while other of Internal positive control (324 bp), while non transgenic samples lack transgene (377 bp).
